# Eight-year post-trial follow-up of morbidity and mortality of telephone health coaching

**DOI:** 10.1186/s12913-021-07263-w

**Published:** 2021-11-15

**Authors:** Erja Mustonen, Iiris Hörhammer, Kristiina Patja, Pilvikki Absetz, Johanna Lammintakanen, Martti Talja, Risto Kuronen, Miika Linna

**Affiliations:** 1 Social and Health Care Reform, Finnish Institute for Health and Welfare, PL 30, 00271 Helsinki, Finland; 2 Healthcare Engineering, Management and Architecture Institute, Aalto University, PL 11000, 00076 Espoo, AALTO Finland; 3 Department of Public Health, University of Helsinki, Yliopistonkatu 4, 00100 Helsinki, Finland; 4 Tampere University, Kalevantie 4, 33014 Tampere, Finland; 5 University of Eastern Finland, Yliopistonranta 1, 70210 Kuopio, Finland; 6 Päijät-Häme Joint Authority for Health and Wellbeing, Keskussairaalankatu 7, 15850 Lahti, Finland

**Keywords:** Health coaching, Type 2 diabetes, Coronary artery disease, Morbidity, Mortality

## Abstract

**Background:**

Health coaching is a patient-centred approach to supporting self-management for the chronic conditions. However, long-term evidence of effectiveness of health coaching remains scarce. The object of this study was to evaluate the long-term effect of telephone health coaching (THC) on mortality and morbidity among people with type 2 diabetes (T2D), coronary artery disease (CAD) and congestive heart failure (CHF)..

**Methods:**

1535 T2D, CAD and CHF patients with unmet treatment targets were randomly allocated into an intervention group (*n* = 1034) and control group (*n* = 501). Intervention group received monthly individual strength-based, autonomy supportive THC sessions (average 30 min) for behavior change with a specially trained nurse for 12 months additional to usual health care. Control group received usual health care services. The primary outcome was a composite of death from cardiovascular causes or non-fatal stroke or non-fatal myocardial infarction (AMI) or unstable angina pectoris (UAP) during a follow-up of 8 years Three other composite endpoints with distinct combinations of fatal and non-fatal cardiovascular events and death from any cause were used as secondary outcomes. Other outcomes followed were the most relevant components of the composite endpoints. Randomized controlled trial (RCT) data was linked to Finnish national health and social care registries and electronic health records (EHR). Post-trial eight-year evaluation was conducted using intention-to-treat (ITT) and per-protocol (PP) analysis.

**Results:**

The composite primary outcome event rate per 100 person years was lower in the intervention group (3.45) than in control group (3.88) in ITT -analysis, but the difference was not statistically significant (hazard ratio in the intervention group 0.87; 95% CI, 0.71 to 1.07; *P* = 0.19). In the subgroup (T2D, CAD/CHF) analysis, there were no statistically significant effects. The secondary PP-analysis showed statistically significant benefits for those who participated in the study.

**Conclusions:**

No statistically significant effect of health coaching on mortality and morbidity was found in intention to treat analysis. The per protocol results suggest, however, that the intervention may be effective among patients who are willing and able to participate in health coaching. More research is needed to identify patients most likely to benefit from low-intensity health coaching.

**Trial registration:**

NCT00552903 (registration date: the 1st of November 2007, updated the 3rd of February 2009).

**Supplementary Information:**

The online version contains supplementary material available at 10.1186/s12913-021-07263-w.

## Introduction 

The burden of chronic disease is a major challenge in health care, especially type 2 diabetes (T2D) and cardiovascular diseases (CVD). It has been estimated that approximately 500, 000 people in Finland (9%) live with T2D. Complications, such as nephropathy, retinopathy and cardiovascular [1, 2] events are common among those with unmet treatment targets and behavioural risk factors, such as hypertension, obesity, smoking, sedentariness and unhealthy diet [3, 4]. Furthermore, T2D is the most prominent risk factor for cardiovascular diseases (CVD), which are the leading causes of the death in the European Union (37.1%) and in Finland (37.5%) [5].

Multifactorial interventions have been shown to be effective in preventing cardiac clinical events among patients with T2D or heart diseases in the short term (two years or less), with an average of 43% relative risk reduction (RR 0.57), but no significant effects have been found on mortality (3,4, 6-). Multifactorial interventions are typically resource-intensive with a very high number of contacts over an extended period, emphasising disease management with medical care rather than self-management by behaviour change, [6] and hence may not be feasible for scaling up or for maintenance by patients in their daily lives.

Health coaching is a patient-centred approach to supporting self-management [7]. It typically relies on motivational interviewing, shared decision-making and collaborative goal setting [8, 9]. Reviews on health coaching have reported mixed findings, including some modest short-term (12–48 months) benefits in terms of a heterogeneous set of psychological, behavioural, physiological and health service utilization outcomes [10–14]. The longest reported follow-up is 6.35 years, during which a significant reduction in overall mortality and total costs for health insurers was found as a result of coaching [15]. However, conclusive evidence on the effectiveness of health coaching is lacking in the long term [10, 16].

The TERVA Health Coaching -trial implemented a low-intensity telephone health coaching (THC) programme for patients with unmet treatment targets for T2D, coronary artery disease (CAD) and congestive heart failure (CHF) in the Päijät-Häme region in southern Finland. It was tested as a randomised controlled trial (RCT) from 2007 to 2009 [17]. At the end of the intervention period of 12 months, a statistically significant difference was reached for diastolic blood pressure but not for other clinical outcomes, such as the glycated haemoglobin (HbA1c) and serum totals and low-density lipoprotein (LDL) cholesterol. The systolic blood pressure and waist circumference target levels were reached more frequently in the intervention group [17]. Over the 12-month intervention period, THC improved patients’ quality of life with moderate costs. Incremental cost-effectiveness ratio of the intervention varied between patient subgroups, being good among T2D patients (€20,000 per QALY), and intermediate among all (€48,000 euros per QALY) [18]. In long-term follow-up, over 8 years, we found that the total social and health care costs were lower in the intervention group. During the first two years costs were higher in THC -group, but after that the costs were lower to the end of the follow-up period [19]. While behavioural changes, such as health coaches’ learning of coaching skills and patients’ lifestyle changes, can take 1–3 years to actualize, the effectiveness of THC -intervention may be delayed [20]. In this study, we report findings on the TERVA trial’s impact on morbidity and mortality among T2D and CAD/CHF patients in an eight-year post-intervention follow-up.

## Methods

### Study design and participants

TERVA was a prospective, longitudinal, 3 × 2 RCT with three disease groups randomised into intervention and control arms. The recruitment of participants has previously been described in detail [17]. In short, 5500 patients were initially identified from electronic patient records in secondary care according to laboratory inclusion criteria: HbA1c > 7 (%) or total cholesterol > 4.5 (mmol/l) or LDL > 2.5 (mmol/l) over six months before inclusion. A research nurse assessed the eligibility according to the final inclusion criteria (e.g., BP > 140/85) and found 2594 patients of whom 1535 (59.2%) gave consent and were randomised to either the intervention or control groups with a 2:1 ratio. Statistical power calculations were conducted for clinical variables in the primary study to verify that the imbalance between study groups was acceptable. The baseline findings in clinical parameters showed no significant differences between the groups [17]. Patients with more than one disease were allocated to their most prominent disease group using the following hierarchy: 1) CHF, 2) CAD/CHF and 3) T2D. In this follow-up study, CHF patient group was combined to the CAD/CHF group [16]. National health care register data was used to report morbidity and mortality among the trial participants in this eight-year follow-up study.

### Care as usual

The participants received routine social and health care [16] based on the national guidelines of care, with typically 2–6 visits per year depending on the state of their disease (The Finnish Current Care Guidelines) [21]. Treatment of CAD and CHF primarily occurs in secondary care, with an additional 1–2 individual follow-up appointments per year in primary care.

### Intervention

In addition to usual care, the intervention group received monthly individual THC. Experienced and specially trained nurses and public health nurses provided the coaching [17], which was autonomy-supportive, utilised motivational interviewing techniques and facilitated self-monitoring, goal setting and action planning for self-management according to the patients’ needs. The coaches focused on eight key recommendations with variation in emphasis based on patient preference: 1) know how and when to call for help, 2) learn about the condition and set goals, 3) take medicines correctly, 4) get recommended tests and services, 5) work to improve or maintain the condition, 6) make lifestyle changes and reduce risk, 7) build on strengths and overcome obstacles and 8) follow up with specialists and appointments. In addition to THC, self-management booklets supported the participants’ progress toward the key recommendations. The coaches utilised a traffic light system for following the patients’ progress. They also had access to the patients’ electronic health records in primary and secondary care and possibility to document health coaching or health status, but they were not fully integrated with the primary care teams [17].

The patients received 10–11 calls for an average of 30 min each during the 12-month intervention period [17]. Because of this short contact time, TERVA was a very low-intensity intervention [6]. To increase the fidelity of the implementation, quality control on the length, frequency and content of the calls was administered [17]. Quality assurance included call reviews identifying and strengthening coaching skills, such as active listening, posing open questions, reflection and summarising the discussion [22].

### Measures

Outcome measures were selected following the Look AHEAD -research group who studied the long-term effects of intensive lifestyle intervention on cardiovascular events among overweight or obese patients with type 2 diabetes [23]. The primary endpoint was the first occurrence of a composite cardiovascular outcome. This included death from cardiovascular (CVD) causes, non-fatal stroke, non-fatal acute myocardial infarction (AMI) and unstable angina pectoris (UAP) [23]. Three secondary composite outcomes were also followed: i) death from cardiovascular causes or non-fatal stroke or non-fatal AMI, ii) death from any cause or non-fatal stroke or non-fatal AMI and iii) death from any cause or non-fatal stroke, non-fatal AMI or UAP, coronary artery bypass grafting (CABG), percutaneous transluminal coronary angioplasty (PTCA) or heart failure or peripheral vascular disease (PVD) [23]. Other outcomes were death (all causes), AMI (fatal or non-fatal), stroke (fatal or non-fatal), renal insufficiency, PVD and hospitalisation due to CHF [23].

### Data

The data for the clinical endpoints from 2007 to 2015 were obtained from the Finnish national registries maintained by the National Institute for Health and Welfare and Statistics Finland. Using a unique personal identification code, we reliably linked the patient cohorts to the registers and retrieved comprehensive data on the dates for all diagnoses (ICD-10), diagnostic and treatment procedures (Nordic Classification of Surgical Procedures), hospital admissions, service contacts in social and health care and deaths for each individual. The registers included the National Discharge Registry,

the National Registry for Primary Care Contact, the Discharge Register for Social Care and the Cause of Death Registry by Statistics Finland.

### Statistical analysis

The similarities between the baseline characteristics in the intervention and control groups were tested using chi-square and t-tests. The Cox proportional hazard regression was used to estimate the risk hazard ratio (HR) of primary and secondary endpoints in the intervention and control groups. The Kaplan-Meier estimator curve was used to report the proportion of patients who had the first event in the primary endpoint, and the Cox regression was used to report HRs and 95% Confidence Interval (CI) for each endpoint. All statistical analyses were conducted using Stata version 15.0.

We report the findings of intention-to-treat (ITT) and per-protocol (PP) analyses [24–26]. The ITT analysis included all patients who had given their informed consent to participation (***n*** = 1535). PP analysis included 1306 patients, who participated in at least one study-related activity. Subgroup analyses were conducted for the T2D and CAD/CHF subgroups.

## Results

The ITT analysis included 1033 patients in the intervention group and 500 patients in the control group. Probably due to emigration, one patient from each group could not be traced in the Finnish national registries and were therefore lost to follow-up. The patient characteristics in the two research groups were similar, with no statistically significant differences between them (Table 1). In the intervention and control groups, the average age of the participants was 65.0 and 65.4 years, respectively, and the proportion of males was 60.6 and 57.8%, respectively. At the eight-year follow-up, 26% of the patients in the intervention and 28% in the control group had died.

**Table 1:** Characteristics of the patients at baseline

Variable	Control group (***N*** = 500)	Intervention group (***N*** = 1033)
Age, mean years	65.4	65.0
Age over 75 years, (%)	16.2	14.1
Male sex (%)	57.8	60.6
Type 2 diabetes, n (%)	355 (71.0)	769 (74.3)
Coronary artery disease, n (%)	97 (19.4)	172 (16.6)
Congestive heart failure, n (%)	43 (8.6)	92 (8.8)
Multimorbidity, (%)	48.0	48.8
Number of chronic conditions, mean	1.67	1.76
Blood pressure (diastolic), mean (n)	84.7 (404)	83.5 ( 812)
Blood pressure (systolic), mean (n)	143.5 (404)	141.9 (812)
Body mass index, mean (n)	25.5 (404)	24.5 (812)
Quality of life (15D), mean (n)	0.861 (470)	0.843 (962)
HbA1c % (n)	7.7 (224)	7.5 (415)
Serum total cholesterol mmol (n)	4.4 (93)	4.3 (250)
Serum high-density lipoprotein mmol (n)	1.26 (93)	1.26 (245)
Serum low-density lipoprotein mmol (n)	2.35 (91)	2.23 (245)

**Fig. 1:**
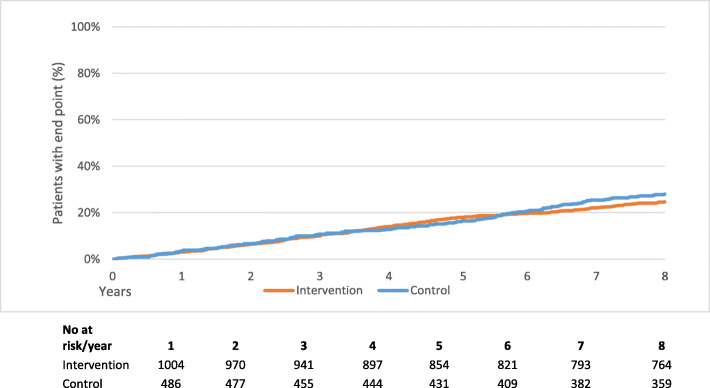
Kaplan-Meier estimates of the cumulative proportion of patients with a primary endpoint event in the intention to treat (ITT) analysis in telephone health coaching. The numbers of patients at risk in each study group in the end of each follow-up year are shown below the graph

**Fig. 2:**
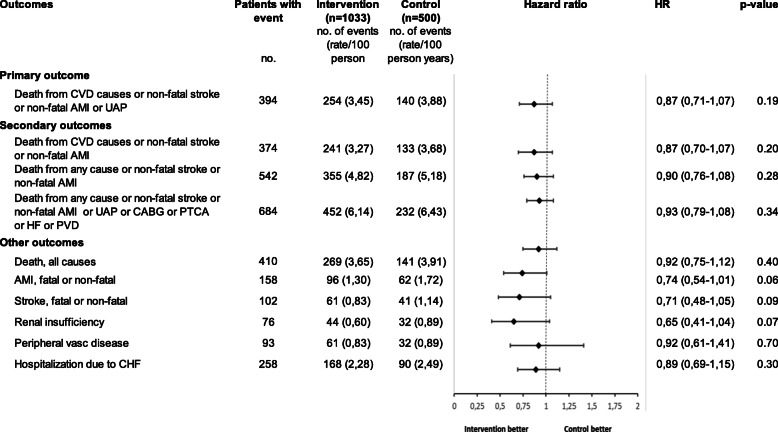
Cox proportional hazard ratios (HR) for the primary, secondary and other outcomes in intention to treat (ITT) analysis. Abbreviations: CVD = cardio vascular disease, AMI = myocardial infarction, UAP = unstable angina pectoris, CABG = coronary artery bypass grafting, PTCA = Percutaneous transluminal coronary angioplasty, PVD = Peripheral vascular disease, CHF = cardiac heart failure

The rate of events per 100 person years was lower in the intervention group for all outcomes, but the differences were not statistically significant compared to the control group. The composite primary outcome – first event of death from CVD causes, non-fatal stroke, non-fatal AMI or UAP – occurred at a rate of 3.45 events per 100 person years in the intervention group and 3.88 events per person years in the control group. The difference in the occurrence between the groups was, however, not statistically significant (HR 0.87; 95% CI, 0.71 to 1.07; *P* = 0.19) (Figs. 1 and 2). There were no significant effects of the intervention on the secondary outcomes or the other outcomes in the ITT analysis, nor did the subgroup ITT analysis show any statistically significant effects among CAD/CHF or T2D patients.

The PP analysis included 853 patients in the intervention group and 453 patients in the control group, excluding those patients who did not perform any activities related to the study. The results indicated some significant differences in the outcomes. Renal insufficiency occurred more often in the control group (0.86 events per 100 person years) than in the intervention group (0.49 events per 100 person years, HR in the intervention group 0.56; 95% CI, 0.34 to 0.94; *P* = 0.02) (Supplement Table 1). In the PP subgroup analysis, statistically significant differences were found among CAD/CHF patients with two outcomes: death from any cause or stroke or AMI or UAP or CABG or PTCA or CHF or PVD (HR 0.73, CI 0.54–0.99, *P* = 0.04) and renal insufficiency (HR 0.35, CI 0.13–0.97, *P* = 0.04) (Supplement Table 2).

## Discussion

In this eight-year follow-up of the TERVA trial, we studied the long-term effects of a 12-month health coaching intervention on mortality and morbidity among patients with T2D or CAD/CHF using comprehensive data from the Finnish health registries. At the baseline, patients were fairly well controlled for the clinical risk factors, and there was relatively little room for improvement. However, events of all cardiovascular mortality and morbidity outcomes were more common in the control group than in the intervention group, but no statistically significant effects of the intervention were found among all study participants or in the T2D and CAD/CHF subgroups.

### Comparison with other studies

To our knowledge, this is the first RCT evaluating the effects of telephone-based health coaching on mortality and morbidity among T2D and CAD/CHF patients in a follow-up extending to 8 years. The follow-up times in health coaching interventions have often been relatively short, with most ranging from 12 to 24 months. Improvements in clinical and behavioural outcomes and risk behaviours have been mixed, and effectiveness over the long term is only speculated [10, 27–29]. Byrnes et al. (2018) found a significant reduction in overall mortality among CVD patients after a six-month telephone coaching with e-mails using a matched-controls RCT with a 6.3-year follow-up [14]. In Angermayr and colleagues’ (2010) review [6] of more intensive multifactorial interventions, the longest follow-ups had not extended beyond 5 years, and often when improvements had been found for BMI, blood glucose, blood lipids or blood pressure, they had not been sustained during the post-intervention follow-up and had no effect on mortality. Ueki et al. (2017) and the Action for Health in Diabetes (AHEAD) - group (2013) reported similar findings: significant improvements of risk factors during the intensive intervention phase but no effect on mortality or morbidity in the long term [23, 29]. It may be that multifactorial target-driven interventions emphasising compliance rather than supporting patient autonomy and competence are not able to produce sustainable changes.

### Strengths and limitations of the study

We conducted our primary intervention study in a real-life setting using an RCT design, with the baseline findings indicating successful randomisation [17]. A major strength of the study was the length of the follow-up; to our knowledge, this study is among the longest follow-up studies of health coaching effects on mortality and morbidity. Also, by using the Finnish national registers with personal identification numbers, we were able to obtain comprehensive, high-quality data on the health care service usage of the participants, allowing us to assess the effects of health coaching on multiple cardiovascular and other severe distal endpoints.

The limitations of this study concern the sample size. First, the sample size of the trial was originally designed to test short-term effects of the intervention and therefore may have been too small to detect significant effects in distal endpoints. This holds to the whole participant group and especially to the subgroup of CAD/CHF, having only 264 and 142 patients in the intervention and control groups, respectively. Second, there was a notable number of patients who dropped out in the early stages of the TERVA trial; 180 patients in intervention group and 47 patients in control group did not attend the initial clinical measurements or any other study-related activities. Dropout was more common in the intervention group (17.4%) than in the control group (9.4%). Dropouts in the intervention group were more frequently diagnosed with more than one of the target conditions (T2D, CAD, CHF) in baseline (52.8 and 40.4% respectively), were younger (mean age 65.1 and 67.9 years respectively) (Supplement Table 3), and had a CVD event (26.5 and 38.3%) or died more rarely (39.8 and 46.8% respectively; HR for death by any cause .79, 95% CI: 0.49 to 1.27) during the follow-up than dropouts in the control group. This comparison suggests some differences in the attrition between the research arms. It also shows that the exclusion of the dropouts in the PP analysis favors the control group rather than the intervention group. In PP analysis we excluded those patients, who did not perform any activities related to the study. It allowed us to make some observations of reduced cardiovascular events among those who participated to the study. In our previous TERVA -trial reporting [19], we found that the 8-year total social and health care costs were lower in intervention group; in ITT -analysis 3% lower (approx. €1200/patient/8-year) and in PP-analysis 14% lower (approx. €6000/per patient/8-year). Although difference was not statistically significant in ITT -analysis, the difference in PP analysis implies that health coaching may be acceptable from cost-effectiveness perspective when improvements in health outcomes are set against reductions in cost of care. From a health services systems perspective, this is important information. and multidimensional evaluation is needed.

### Conclusion 

Health care policy makers should consider the time lag between programme implementation, patients’ behavioural and physiological risk factor changes and health gains and reduced cost. The conservative estimates regarding all participants in this study showed no definitive evidence of the effects of health coaching on mortality or morbidity among T2D and CAD/CHF patients. Better identification of those patients able to benefit from health coaching may aid in allocating coaching efforts in an effective way. Further, more multidisciplinary and multidimensional research is needed to understand the effectiveness of complex self-care interventions such as health coaching.

## Supplementary Information

**Additional file 1.**

## Data Availability

Access to these databases is closed and permission to use the data was received from the national authorities.

## Abbreviations

AMI: Myocardial infarction
CABG: Coronary artery bypass grafting
CAD: Coronary artery disease
CHF: Congestive heart failure
CI: Confidence interval
CVD: Cardiovascular disease
HbA1c: Glycated haemoglobin
HDL: High density lipoprotein
HR: Hazard Rate
ICER: Incremental Cost Effectiveness Ratio
ITT: Intention-to-treat
LDL: Low-density lipoprotein
PP: Per protocol
PTCA: Percutaneous transluminal coronary angioplasty
PVD: Peripheral vascular disease
QALY: Quality Adjusted Life Years
RCT: Randomized controlled trial
S-kol: Serum total cholesterol
TERVA: Telephone-based health coaching development and research program
THC: Telephone Health Coaching
T2D: Type 2 diabetes
UAP: Unstable angina pectoris
