# Supplementary material for: Eight-year post-trial follow-up of morbidity and mortality of telephone health coaching
Source: BMC Health Serv Res. 2021 Nov 15;21:1237. doi: 10.1186/s12913-021-07263-w (PMC8594149; doi:10.1186/s12913-021-07263-w)
Supplement: Supplementary file 1 — Additional file 1. [file 12913_2021_7263_MOESM1_ESM.docx]

**Supplement files**

Supplement Table 1. Cox proportional hazard ratio (HR) of all patients with event and p-values comparing the primary, secondary and other outcomes between the intervention and control groups in the Per Protocol - analysis.

Supplement Table 2. Cox proportional hazard ratio (HR) of coronary artery disease (CAD) patients with event and p-values comparing the primary, secondary and other outcomes between the intervention and control groups in the Per Protocol -analysis.

Supplement Table 3. The baseline characteristics for ITT, PP and dropout -patients in intervention and control groups (age, proportion of age over 75, gender, prevalence of T2D, CAD and CHF), proportion of those diagnosed for more than one of T2D, CAD, CHF and number of chronic conditions.

|  | ITT  interventio (N=1033) | ITT  control (N=500) | PP  intervention  (N=853) | PP  control  N=453 | Dropout intervention  (N=180) | Dropout  control  (N=47) |
| --- | --- | --- | --- | --- | --- | --- |
| Age, mean years | 65.0 | 65.4 | 64.9 | 65.1 | 65.1 | 67.9 |
| Age over 75 years, (%) | 14.1 | 16.2 | 12.7 | 15.0 | 21.1 | 27.6 |
| Male sex (%) | 60.6 | 57.8 | 61.8 | 58.5 | 56.1 | 61.7 |
| Type 2 diabetes n (%) | 769 (74.3) | 355 (71.0) | 637 (74.7) | 324 (71.5) | 132 (73.3) | 35 (74.4) |
| Coronary artery disease, n (%) | 172 (16.6) | 97 (19.4) | 145 (17.0) | 90 (19.8) | 27 (15.0) | 7 (14.9) |
| Congestive heart failure, n (%) | 92 (8.8) | 43 (8.6) | 44 (8.8) | 39 (8.6) | 21 (11.6) | 5 (10.6) |
| Diagnosed for more than one of T2D, CAD, CHF, n (%) | 504 (48.8) | 240 (48.0) | 410 (48.1) | 220 (48.7) | 95 (52.8) | 19 (40.4) |
| Number of chronic conditions (mean) | 1.76 | 1.67 | 1.72 | 1.66 | 2.0 | 1.78 |
